# Applicability of Aerosol Deposition Process for flexible electronic device and determining the Film Formation Mechanism with Cushioning Effects

**DOI:** 10.1038/s41598-019-38477-y

**Published:** 2019-02-15

**Authors:** Chuljun Lee, Myung-Yeon Cho, Myungjun Kim, Jiyun Jang, Yoonsub Oh, Kihoon Oh, Seunghyun Kim, Byungwook Park, Byungkwan Kim, Sang-Mo Koo, Jong-Min Oh, Daeseok Lee

**Affiliations:** Department of Electronic Materials Engineering, Kwangwoon University, 20 Kwangwoon-ro, Seoul, 01897 Republic of Korea

## Abstract

In this paper, we demonstrated the feasibility of the Aerosol Deposition (AD) method which can be adapted as a future fabrication process for flexible electronic devices. On the basis of this method’s noticeable advantages such as room-temperature processing, suitability for mass production, wide material selectivity, and direct fabrication on a flexible substrate, we fabricated and evaluated a flexible conductive bridge random access memory (CBRAM) to confirm the feasibility of this method. The CBRAM was fabricated by the AD-method, and a novel film formation mechanism was observed and analyzed. Considering that the analyzed film formation mechanism is notably different with previously reported for film formation mechanisms of the AD method, these results of study will provide strong guidance for the fabrication of flexible electronic device on ductile substrate.

## Introduction

The Aerosol Deposition (AD) method is a deposition process, which has been recently and rarely introduced in electronic device field. The AD method can be used to deposit non-metal, metal, thin and thick layers by spraying solid powder. This deposition process has several advantages such as room temperature processing, suitability for mass production, direct deposition and low-cost process^1–3^. Moreover, the AD method is an attractive deposition process for the formation of composite films due to its unique and simple deposition mechanism, which sprays solid-state raw powder such that the composite layer can be formed by spraying mixed powder at the substrate^4^. According to previous studies, various composite films such as ceramic-ceramic^4^ ceramic-polymer^5^ and ceramic-metal^6^ have been reported, which also shows wide material selectivity of AD method. Exploiting these desirable process properties, researchers have utilized the AD method for fabrication in various application such as surface coating, sensor devices, fuel cells, optical device, etc; Table 1 lists the applications, utilized materials, references to reports in the literature for the AD method, demonstrating the wide applicability of the AD method. Therefore, the advantages and wide applicability of AD method show that the AD method can be applied to other applications, as well.

**Table 1:** Various applications based on the AD method.

Application		Material	Reference
Surface coatings	Surface protecting	YSZ	^19^
	Biocomponent	Hydroxyapatite (HA), Brushite	^20, 21^
Sensor devices	Gas sensor	ZnO, SrTi_0.7_Fe_0.3_O_3−δ_	^22, 23^
	Thermistor	NiMn_2_O_4_	^24^
	Humidity sensor	BaTiO_3_	^8^
Fuel cells	Buffer layer	(Gd_0.1_Ce_0.9_)O_2−δ_(GDC)-Gd_2_O_3_	^25^
	Electrolyte	BaZr_0.8_Y_0.2_O_3−δ_	^26^
Optical devices	Electro-optical modulator	PLZT	^27^
	Optical fiber electric field sensor	PZT	^28^
Passive component	Embedded capacitor	BaTiO_3_	^29^

Moreover, the AD method is attractive as the fabrication process of flexible electronic applications because of its desirable process characteristics such as room-temperature processing (for a flexible substrate with low melting point), suitability to mass production, and low-cost process. However, despite these characteristics, AD method based flexible electronic applications have rarely been researched. Thus, we fabricated the flexible electronic device to confirm the feasibility of applying the AD to the fabrication process for flexible electronic applications.

In this research, for the first time, we developed a flexible two-terminal Non-Volatile Memory (NVM) by utilizing the AD method. As a NVMs device, we fabricated metal-insulator-metal structured Conductive Bridge Random Access Memory (CBRAM); this memory has an operation mechanism in which metal conducting path within the solid electrolyte layer is created (or dissolved) by an electrochemical reaction (oxidation and reduction) of the metal^7^. On the basis of this mechanism, we fabricated AD method based CBRAM (AD-CBRAM) with an Al_2_O_3_/Ag composite layer (active layer: the electrochemical reaction is occurred by applied electric field) and the Al_2_O_3_/Ag composite layer was deposited by the AD method without the thermal process. Consequently, we successfully obtained the NVM characteristics of flexible AD-CBRAM, proving a convincing demonstration of the feasibility of applying the AD method to the fabrication of NVM devices, as well as flexible electronic applications.

Furthermore, we observed a novel phenomenon in the deposited Al_2_O_3_/Ag composite layer on the ductile substrate. This phenomenon was not explained by the previously reported mechanism of the AD method. According to the typical AD method mechanism, the firstly deposited layers become more dense than the subsequently deposited layers (hammering effect)^8,9^. By contrast, in the fabricated Al_2_O_3_/Ag composite film, the firstly deposited layers were less dense than the subsequently deposited layers. The finding can be considered to be due to the effects of the flexible substrate, which can provide strong guidance for the fabricating AD method based flexible electronic devices.

## Experiment

### Preparation of Al_2_O_3_/Ag composite powder

For operation of CBRAM, composite layer consisting of metal (Ag) where the electrochemical reaction is occurred by an applied electric field and an electrolyte layer (Al_2_O_3_) was used. To deposit the Al_2_O_3_/Ag composite layer, a mixture of 99.8 wt% of Al_2_O_3_ and 0.2 wt% of Ag powder was used as a starting powder (powder to be sprayed). The Scanning Electron Microscopy (SEM) images and particle size distribution for each powder (Al_2_O_3_, Ag and Al_2_O_3_/Ag composite powder) are shown in Supplementary Information (Fig. S1), as well as the distribution of Ag in composite powder is also observed by Energy Dispersive Spectrometer (EDS) mapping analysis. After mixing these two powders, the starting powder was dried for 24 hours at 155 °C in order to prevent the aggregation of powder by removing the residual moisture.

### Fabrication device

**Figure 1:**
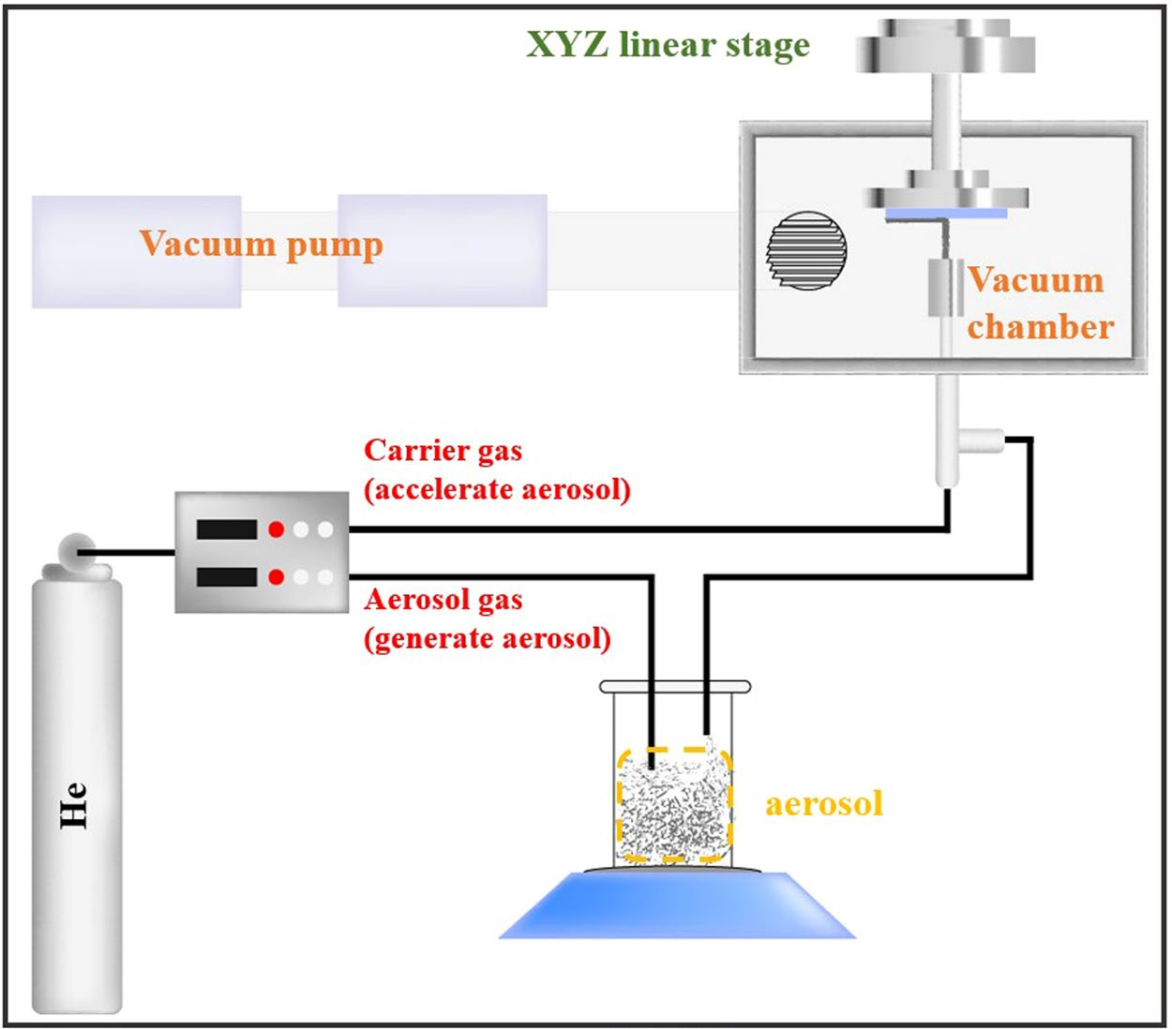
Schematic illustration of key components (vacuum system, gas controller and XYZ  linear stage) of AD system.

The AD method has a simple deposition mechanism in which the targeted film is formed by directly bombarding the accelerated starting powder onto the substrate. To operate this AD system, we constructed some of the key parts, such as the vacuum system, gas controller and x-y linear stage; the overall experimental setup is shown in Fig. 1. First, to prevent contamination and deceleration of the accelerated aerosol (floated starting powder) leaving through the nozzle, the base pressure of the chamber was adjusted to 0.07 Torr. Next, we utilized the non-reactive helium gas to generate and accelerate the aerosol consisting of the Al_2_O_3_/Ag composite powder. The gas flow of the aerosol gas (for aerosol generation) and carrier gas (for aerosol acceleration) were maintained at 4–6 L/min and 10 L/min, respectively, by gas controller. Next, by colliding the accelerated aerosol with a high kinetic energy onto the flexible aluminum-substrate, the Al_2_O_3_/Ag composite film is deposited on the flexible substrate. A detailed description of other parameters, such as nozzle orifice size, scan speed, thickness of composite layer and etc., are provided in the Supplementary Information (Table S1).

The mechanism of CBRAM is that a metal conducting path is formed (or dissolved) by an electrochemical reaction which is occurred by electric field between the two electrodes. Thus, to generate the electric field at the composite layer, aluminum (substrate) and nickel were utilized as the bottom and top electrodes, respectively. The nickel electrode with the thickness of 50 nm was evaporated on the Al_2_O_3_/Ag composite layer, and it was patterned in square shape with the dimension of 300 µm × 300 µm.

## Characterization

The cross-sectional image of the Al_2_O_3_/Ag composite layer was analyzed by Transmission Electron Microscopy (TEM, JEM-2100F, Japan). In the Al_2_O_3_/Ag composite layer, to reveal the differences in the grain size between three different regions (bottom, middle, top), we evaluated the diffraction strength in each region by observing Selected Area Electron Diffraction (SAED) patterns. Moreover, EDS based elemental analysis was used to verify the distribution of silver in the composite layer.

To evaluate the electrical properties of the flexible AD-CBRAM, current-voltage (I–V) characteristics, time-dependent variation and bending-dependent variation were evaluated using a Keithley 2450 source-meter. For the I–V characteristics measurements, consecutive voltages with the interval of 100 mV were induced at the device and the current level was read. Moreover, the limit current (maximum current applied to the device, limited by the equipment) was set to 100 µA to prevent the permanent breakdown of the device. For the time-dependent and bending-dependent variation measurement, we identified whether the two resistance states (high resistance state and low resistance state) of the device was stably maintained under the time and bending stress. To induce bending stress at the device, we utilized convex shaped supporting molds with radius varying from 70 mm to 20 mm; a larger bending stress induced on the device with decreasing radius of the supporting molds.

## Result and Discussion

### Film formation mechanism on the high ductile substrate (cushioning effect)

**Figure 2:**
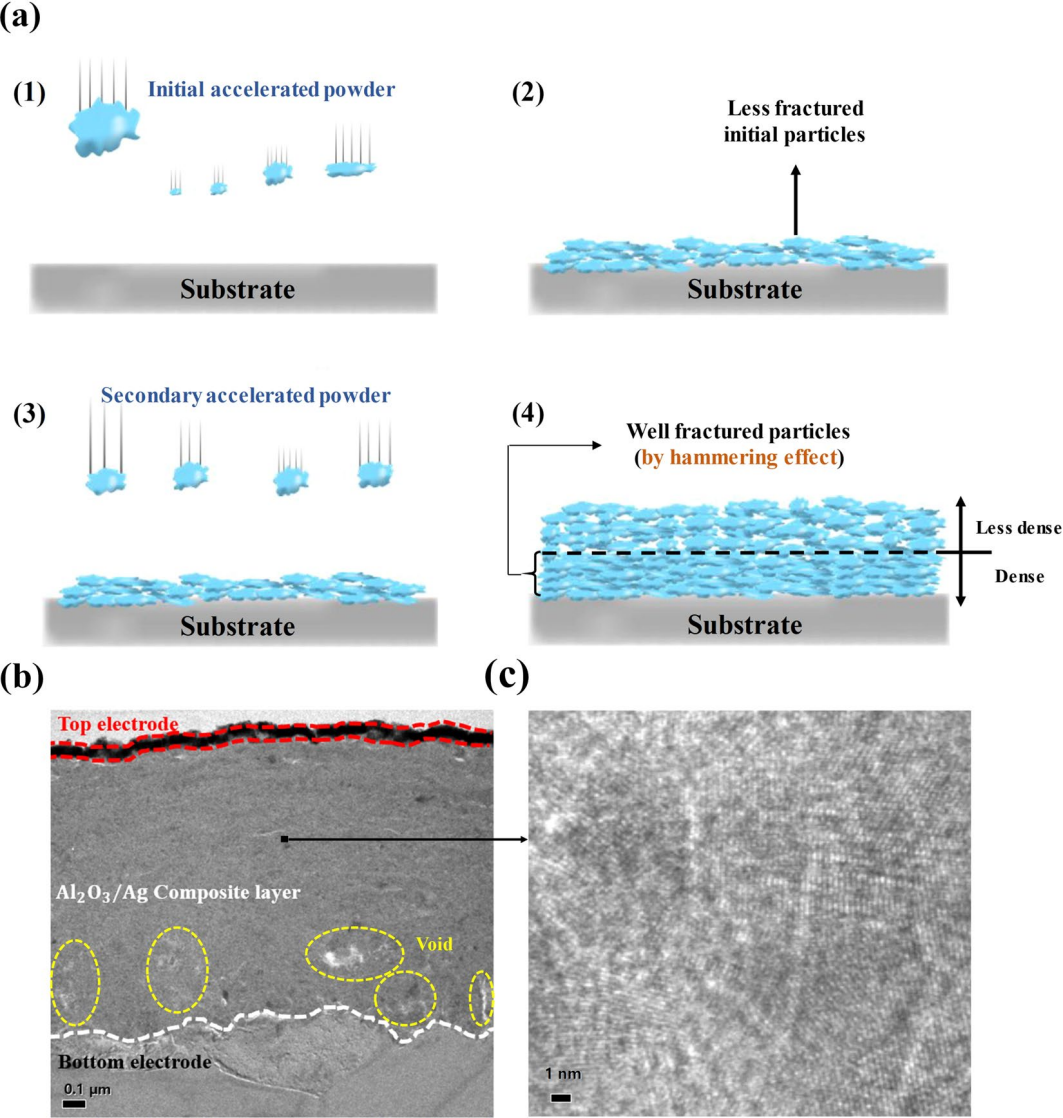
(**a**) Schematic illustration of typical film formation mechanism of AD method (with hammering effect). TEM cross-sectional image for AD-CBRAM (**b**) overall structure (bottom electrode-composite layer-top electrode) (**c**) middle region of composite layer; nanosized crystallites were observed.

On the basis of this non-thermal driven mechanism of AD method^1–3,9^, we deposited the Al_2_O_3_/Ag composite layer without any thermal process; it is attractive for fabrication process of flexible applications due to wide selectivity of substrate. Figure 2(b) shows the cross-sectional TEM image of fabricated AD-CBRAM, which demonstrates that the composite layer having uniform thickness is formed on the aluminum substrate. Moreover, the nanosized crystallites were observed in the middle region of composite layer, which shows fundamental deposition method (using collisions and fragmentation of particles) of the AD method; it is shown in Fig. 2(c).

According to previous studies, the first-deposited layer (bottom region) is more dense than the subsequently deposited layer due to the dominant hammering effect. Figure 2(a) shows previously reported typical film formation mechanism of AD method. First, the initial accelerated particles collide with substrate, which lead to the fragmentation of particles and formation of film. However, although the firstly deposited layer has less dense characteristics due to less fractured initial particles, subsequent particle collisions make the first deposited film dense. This densification of the film occurs by the consistent size reduction of the fractured particles due to the continuous collision of accelerated particles^10^: hammering effect. On the basis of this order, the bottom region of deposited film is denser than upper region.

**Figure 3:**
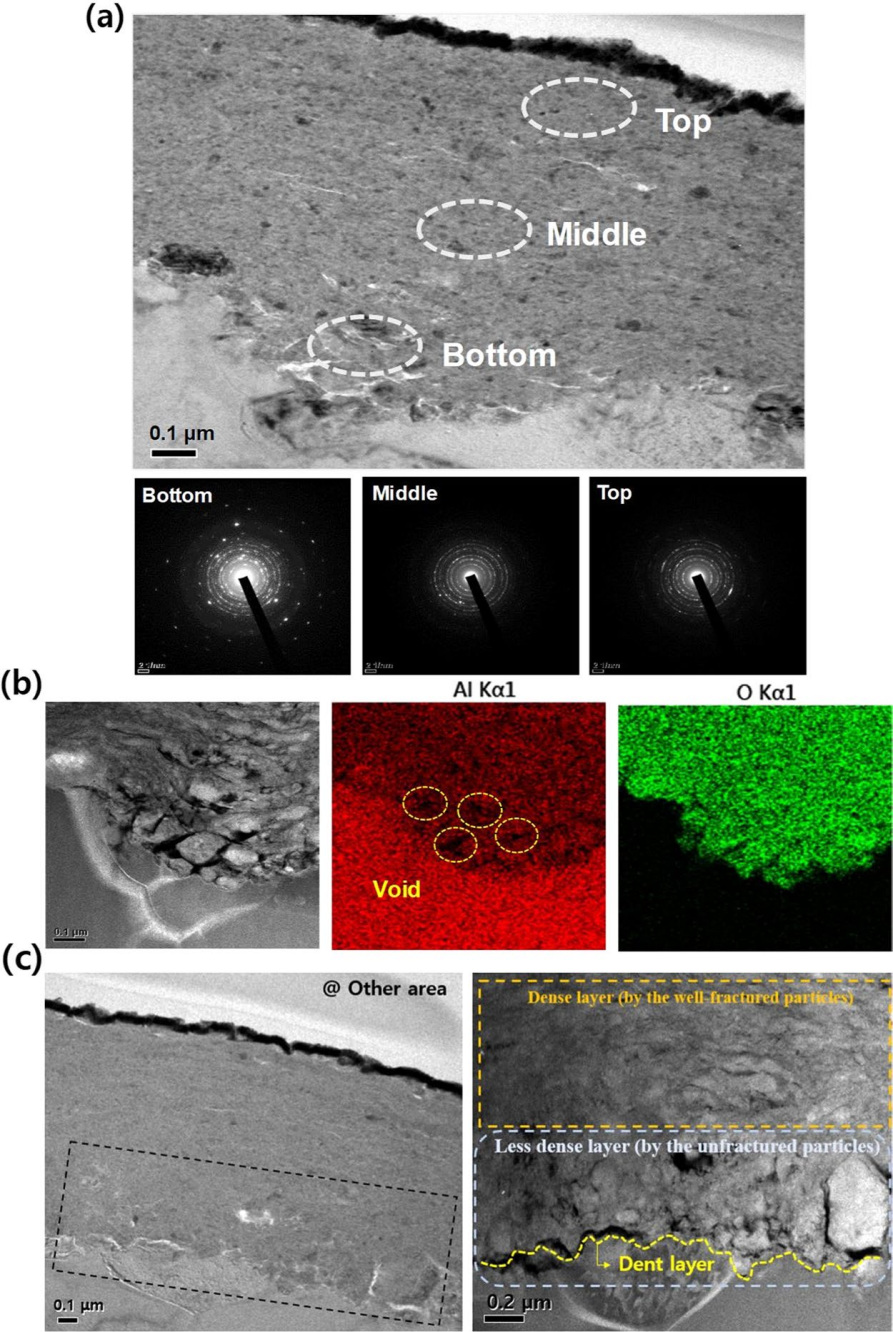
(**a**) Cross-sectional TEM image and SAED patterns for three regions (bottom, middle, top) of composite layer (**b**) STEM and EDS mapping image near the interface composite layer and substrate (**c**) Cross-sectional TEM in other area and STEM image of the region indicated in TEM image.

Interestingly, in the fabricated AD-CBRAM, a phenomenon different from typical film formation mechaism (following the hammering effect) was observed. Figure 2(b) strongly show that the top and bottom region of the composite film have different structural properties; bright spots (less dense area) are concentrated at the bottom region. Moreover, this tendency is also observed throughout the bottom region, which is shown in Fig. 3(a,c) and supplementary information (Fig. S2). Therefore, to investigate this phenomenon, we analyzed the composite layer in detail.

Figure 3(a) shows a cross-sectional TEM image and Selected Area Electron Diffraction (SAED) patterns for the three regions (bottom, middle, top region of composite layer) of the composite layer. The TEM images (including Figs 3(c) and S2) show that the bright spots were distributed at the bottom region. Interestingly, it can be observed from the SAED patterns that stronger and more asymmetric diffraction is detected at the bottom region compare to the upper region. These results demonstrate that the large sized crystallites are disorderly distributed at the bottom region; this phenomenon is also observed at the other area, as shown in supplementary information (Fig. S3).

To clearly investigate this phenomenon, we first conducted a Scanning Transmission Electron Microscopy (STEM) analysis, the results of which are shown in Fig. 3(b,c). From the STEM image, we obtained a definite image for the bottom region, and the less-fractured particles were clearly observed. Thus, considering that the sufficiently fractured particles lead to the densification of the film^10^, the less-fractured particles lead to a less dense film and the presence of voids due to particle boundaries. Moreover, the EDS mapping image in Fig. 3(b) clearly show the voids where no components were detected.

This novel phenomenon may strongly depend on the difference in the mechanical properties of the substrate and the powder. Figure 3(b,c) show not only the formation of a dent layer at the interface composite layer and substrate, but also that the less fractured particles are located in the dent layer. These results strongly imply that the deformation of the substrate, rather than the fracture of the particles, is dominant when the particles collide with the substrate, which is attributed to the difference between the mechanical properties of the particle (high hardness) and the substrate (high ductility). In other words, the substrate having ductility serves to relieve the impact energy when the accelerated particles collide with the substrate. To clearly confirm the dependence for the mechanical property of the substrate, we deposited Al_2_O_3_/Ag composite film on the glass substrate having a hardness larger than Al substrate (with same deposition-conditions). Then, the cross-sectional TEM and STEM analysis were conducted, which shown in supplementary information (Fig. S4). Unlike composite film on the Al substrate having ductility, dense layer consisting of well-fractured particles was clearly observed at the bottom region. It can be explained by sufficient impact energy for fracturing of the particles due to the high hardness of the glass substrate. On the basis of these results, for the first time, we propose that a cushioning effect of the highly ductile substrate exists and can generate the lesser fragmentation of the initial collided particles (near the substrate), as well as reducing the hammering effect by dispersing the kinetic energy of the subsequently collided particles.

**Figure 4:**
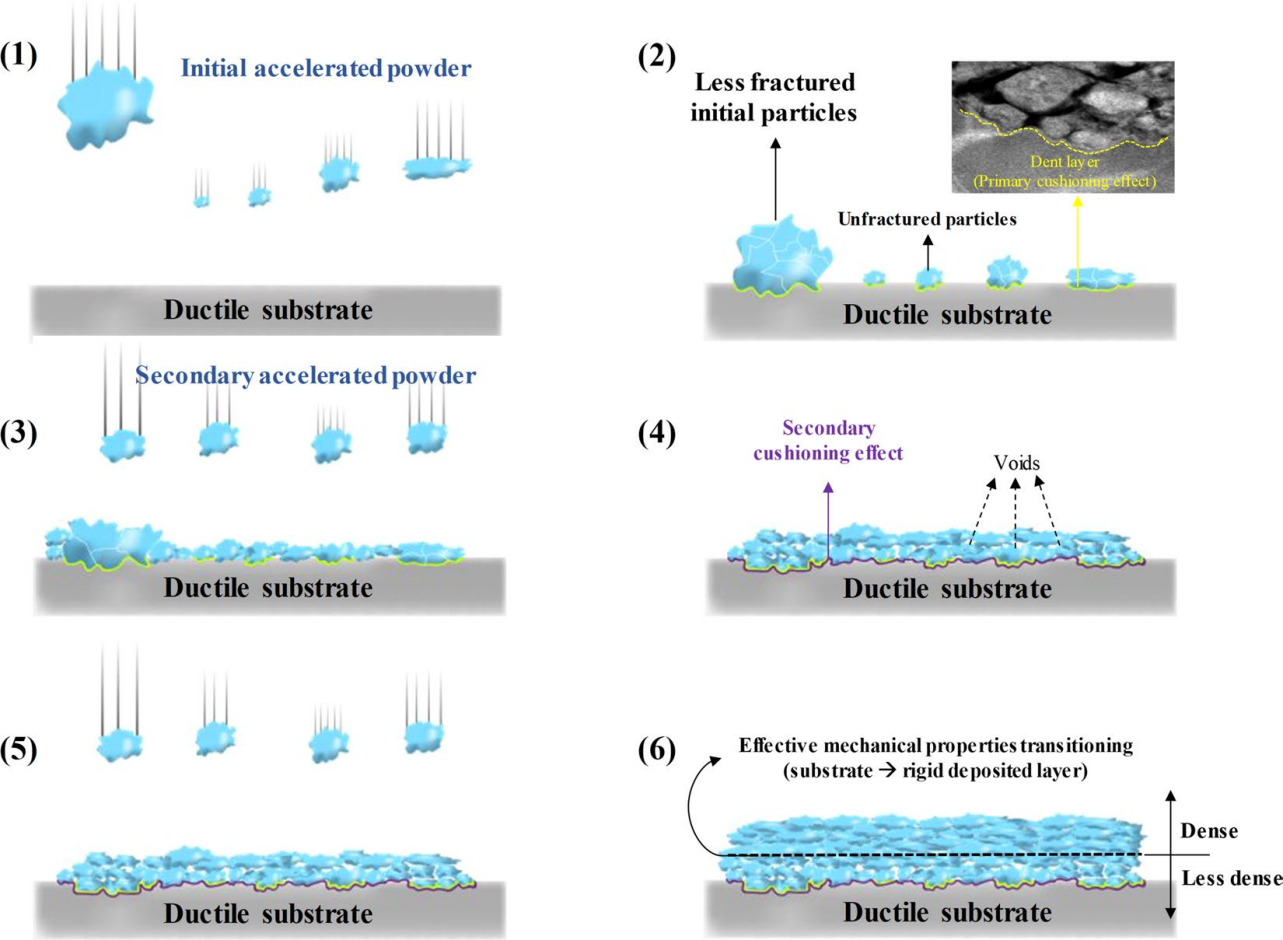
Deposition mechanism on ductile substrate.

Based on cushioning effect of ductile substrate, we propose a novel mechanism for the film formation on the ductile substrate by the AD method; the schematic illustrations are shown in Fig. 4. First, particles accelerated by carrier gas travel to the ductile substrate and collide. However, due to the high ductility of substrate, less (or no) fragmentation of the particles occurs and it is the main cause of the dent layer formation: we refer to this phenomenon as the primary cushioning effect. Second, although the particles subsequently collide with the initial fractured particles near the substrate, sufficient fragmentation of the particles does not occur because the mechanical properties of substrate are dominant. Therefore, this effect limits the fragmentation of the initial less-fractured particles and the secondary accelerated particles: this phenomenon is the secondary cushioning effect. Therefore, the formation of voids and a less-dense layer is mainly attributed to the primary and secondary cushioning effects. Third, the cushioning effect is relieved above a certain thickness of the bottom regions. As a result, a dense layer consisting of well-fractured particles was formed at the middle and top regions, as is clearly demonstrated in Figs 3(a,c) and S3. The reduction of the cushioning effect may be attributed to the transition of the effective mechanical properties of collision surface from the ductile substrate to the rigid Al_2_O_3_/Ag composite layer. As a result of gradual change, the layers of the bottom region become less dense than those of the upper region, representing a unique film formation mechanism that is unlike that of typical AD method. Therefore, the first proposed film formation mechanism considering the cushioning effect of the ductile substrate will provide an effective guideline for the fabrication of applications on the ductile substrate using the AD method.

### Operation of flexible AD-CBRAM

**Figure 5:**
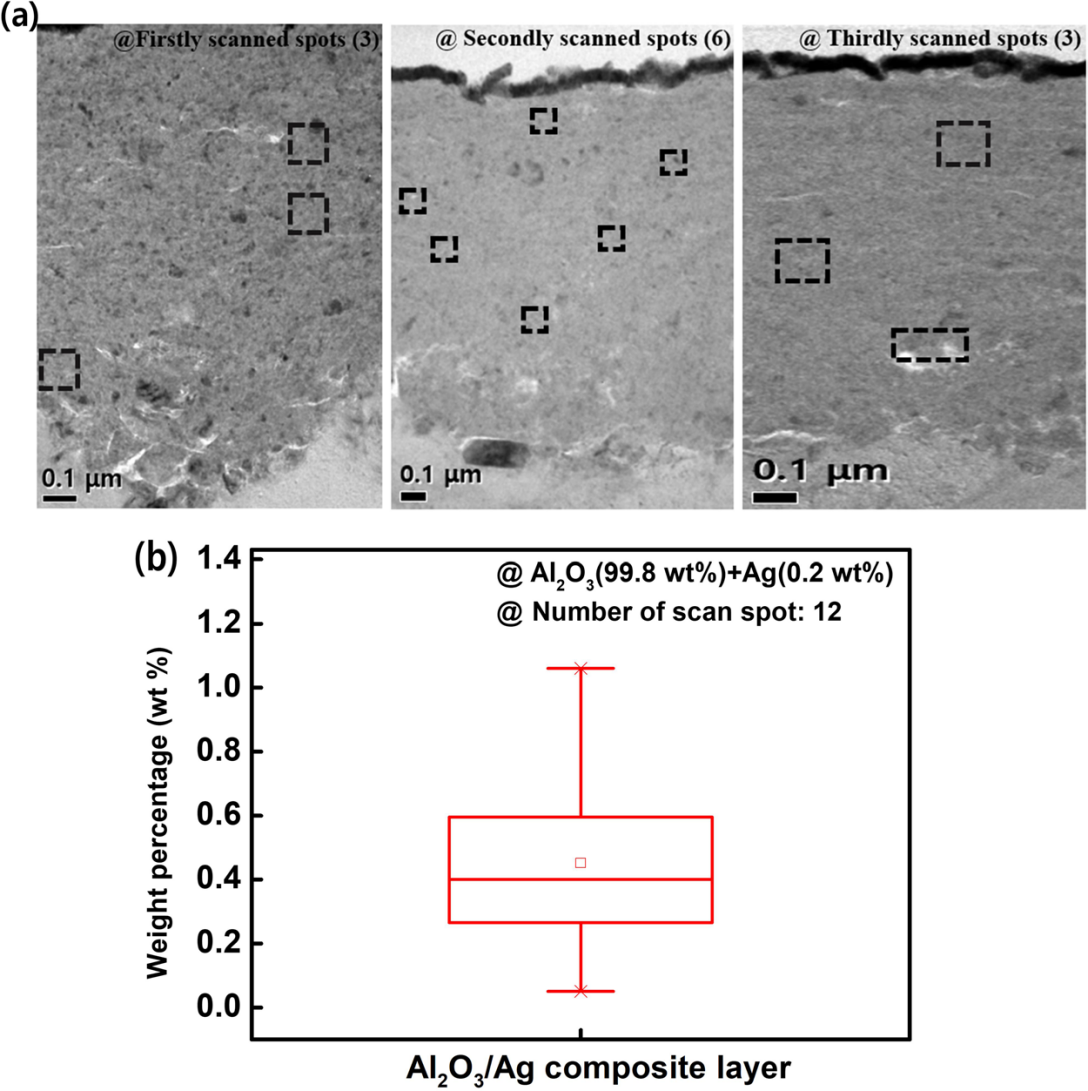
(**a**) Scanned spot regions for EDS spot mapping (**b**) average weight-percentage distribution of silver in the scanned composite layer.

The operational mechanism of CBRAM is based on the metal conducting path being formed (or dissolved) by the electrochemical reaction of a high field-induced-diffusivity metal. Typically, silver or copper are used as the active metal in CBRAM due to their high field-induced-diffusivity^11^. Thus, we used an Al_2_O_3_/Ag composite layer as the active layer in which the electrochemical reaction of silver is occurred by the applied electric field. Figure 5(b) shows the weight percent of silver in the deposited Al_2_O_3_/Ag composite layer evaluated by randomly scanning 12 spots using the EDS component analysis; the randomly scanned spots are shown in Fig. 5(a). It was found that the average weight percentage of silver in the composite film was approximately 0.41 wt% and that silvers are widely distributed in the layer.

The distributed high field-induced-diffusivity metal within the active layer can form (or dissolve) the conducting path by migrating in the layer, leading abrupt transition of the resistance of the active layer^12,13^. According to a previous study, silver doping dependence in the solid electrolyte layer was clearly demonstrated by confirming the resistance transition of the device^13^. Therefore, to confirm the silver dependence in the AD-CBRAM, we compared the electrical properties of the non-composite (pure Al_2_O_3_) layer device and Al_2_O_3_/Ag composite layer device.

**Figure 6:**
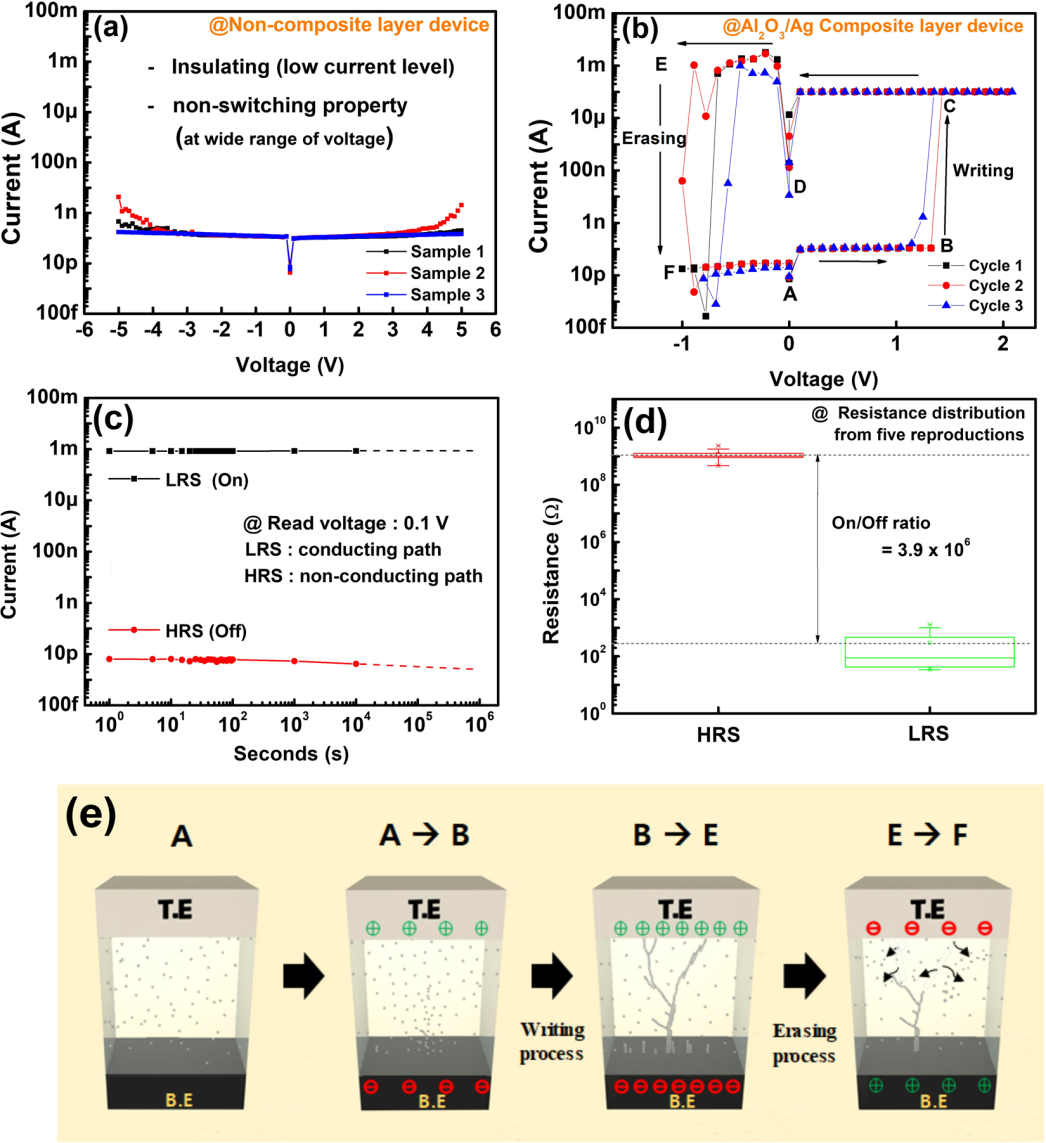
I–V characteristics for (**a**) non-composite layer device and (**b**) composite layer device. (**c**) Time-dependent variation (for HRS, LRS) at room temperature. (**d**) Resistance distribution (HRS and LRS) for five reproductions. (**e**) Schematic illustration of operation mechanism for AD-CBRAM, which related with operation of (**b**).

We first evaluated the electrical properties of the non-composite (pure Al_2_O_3_) layer device; it is shown in Fig. 6(a). It was found that despite the external bias (from 0 V to +5 or −5 V) applied to the device, the abrupt transition of resistance did not occur (insulating behavior was maintained), indicating that no conducting path was formed in the layer. This result is caused by absence of silver with its high field-induced diffusivity. As a next step, we evaluated the electric properties of the Al_2_O_3_/Ag composite layer device and clearly confirmed its operation as a NVM device. Figure 6(b) shows the I–V characteristics of the Al_2_O_3_/Ag composite layer device with consecutive two types of voltage sweep (positive voltage sweep: A→B→C→D and negative bias sweep: D→E→F→A). Consequently, the operation of NVM device (CBRAM) is successfully confirmed on the Al_2_O_3_/Ag composite layer device, and it clearly shows that the distributed Ag in the device is critical parameter for AD-CBRAM. On the basis of the Ag-dependence and many researches for CBRAM^14–16^, we present the operating procedure and mechanism of the AD-CBRAM for the sweep sequence (A→F), which is shown in Fig. 6(e).

At the low positive bias (A→B), the AD-CBRAM has insulating characteristics (HRS: High Resistance State) since the conducting path has not yet formed in the composite layer. However, above about 1.3 V (B: writing voltage which lead to abrupt resistance-transition), the resistance state was abruptly changed from insulating characteristics (HRS) to metallic characteristics (LRS: Low Resistance State). The abrupt transition can be explained by the electro-forming of the Ag conducting path and this resistance-transition process (from HRS to LRS) is called the ‘writing process’ (B→C). After the writing process, the device continuously maintained the LRS characteristics until the positive voltage sweep is over.

Next, the negative voltage (D→E→F→A) was sequentially applied at the device. At small negative bias (below −0.5 V), the LRS result from the writing process consistently remained, clearly indicating the non-volatile characteristics of AD-CBRAM. In other words, although the external bias was removed and a small negative bias (D→E) was applied, the device remembered the previous resistance state (LRS); this memory is attributed to the maintenance of silver conducting path within the composite layer. However, when a larger negative bias was applied (E→F), the resistance state is abruptly changed from LRS to HRS (‘erasing process’). The abrupt resistance-increasing can be explained by rupture of the conducting path. According to previous studies, concentrated electric field and joule-heating at the extremely thin conducting path are critical internal stresses to dissolve the conducting path^17,18^; joule-heating occurs by charge-flow (current) through the atomic-scaled conducting path. Therefore, on the erasing process for AD-CBRAM, the electric field and a high-current (>1 mA) through the conducting path would cause rupture of the conducting path; it leads to electrochemical reaction and joule-heating. Then, the HRS is maintained even after the negative sweep is concluded. These operations (the writing and erasing processes) were also observed in subsequent voltage cycles.

To clearly confirm the NVM characteristics of the AD-CBRAM, we evaluated the time-dependent variation of LRS and HRS, respectively; it is shown in Fig. 6(c). The time-dependent variation is evaluated from the modulation of the HRS and LRS with time. To determine the resistance state of device, the current level was read by applying specific read voltage (0.1 V). Consequently, the current levels of the device in the HRS and LRS are 10 pA (HRS: insulating characteristic) and 1 mA (LRS: metallic characteristic), respectively. Moreover, the HRS and LRS of device were stably maintained for 10^4^ seconds, clearly showing the non-volatile characteristics of resistance state.

As a next step, to confirm the reproducibility for the AD-CBRAM, the two resistance states (HRS and LRS) of reproductions were evaluated, as shown in Fig. 6(d). Overall, the average resistances of the HRS and LRS were 1 GΩ and 300 Ω, respectively. The large difference between two resistance states (about 3.9 × 10^6^ times) is attractive characteristic for the NVM; it is obviously enough on/off margin to store information (‘1’ or ‘0’).

Briefly, we first evaluated the silver dependence by comparing to the non-composite dependence and to the Al_2_O_3_/Ag composite device. Consequently, at the composite layer device, we confirmed that the resistance states (LRS or HRS) of the device can be selectively programmed by external bias with two polarities, which demonstrates the overall operation of AD-CBRAM. Moreover, the programmed two resistance states are stably maintained over time, which strongly demonstrate the non-volatile characteristics of the AD-CBRAM.

**Figure 7:**
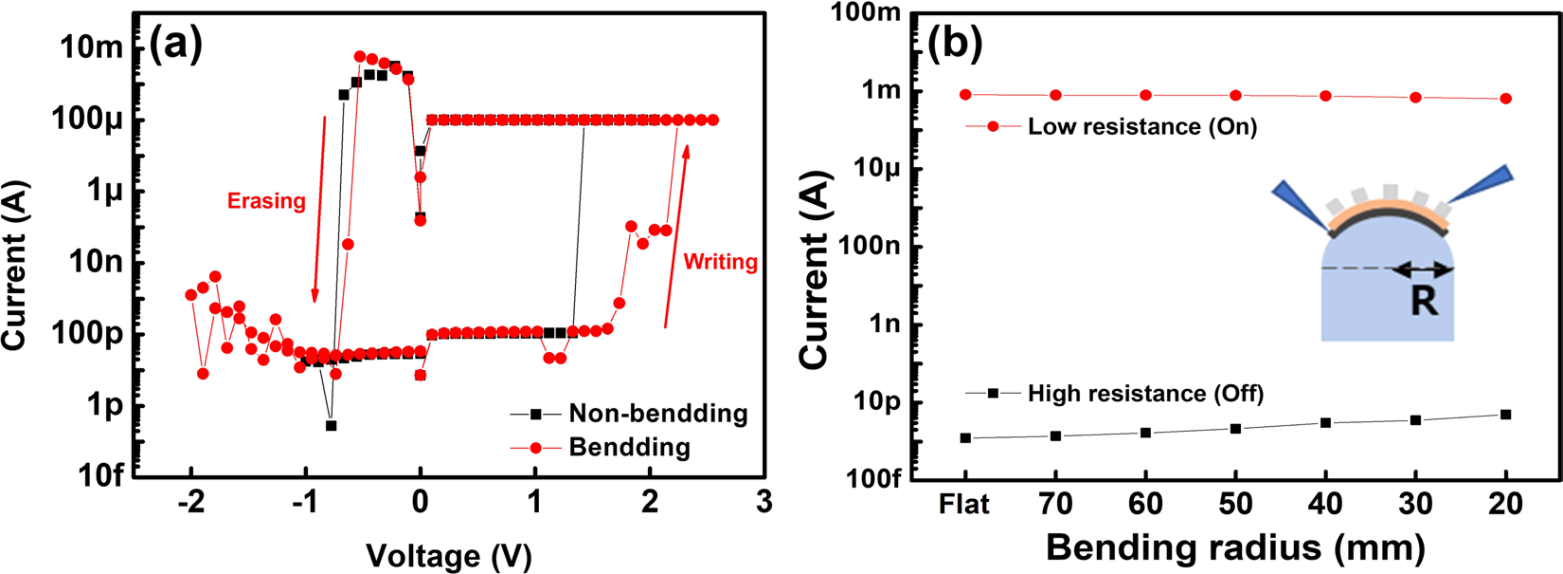
(**a**) I–V characteristics under bending stress (R = 20 mm) (**b**) bending-dependent variation (for HRS, LRS) measurements under the various bending stresses.

Furthermore, we also evaluated the operation of the AD-CBRAM under bending stress conditions. To apply the bending stress, convex shaped supporting molds with radius varying from 70 mm to 20 mm were used. Figure 7(a) shows the I–V characteristics under the two conditions (non-bending stress and bending stress), and for supporting mold with the radius of 20 mm utilized for bending stress. Under the bending stress, even though the writing process condition was slightly changed, the resistance transition from HRS (or LRS) to LRS (HRS) of the device is successfully occurred by the external bias: the resistance states (HRS or LRS) can be programmed by the applied electric field. Moreover, we also evaluated the bending-dependent variation of the AD-CBRAM, which was sequentially measured changing from flat to 20 mm (bending radi-us). Despite the sequential stress, the LRS and HRS are successfully maintained as shown in Fig. 7(b). Thus, these results show the writing (and erasing) operation and NVM characteristics of programmed resistance states under the various bending stresses, indicating that the flexible AD-CBRAM is operated successfully.

## Conclusion

In summary, to demonstrate the feasibility of the AD method for the flexible electronic devices, we developed and evaluated the flexible CBRAM with an Al_2_O_3_/Ag layer by the AD method for the first time. The resistance states (HRS and LRS) of the AD-CBRAM can be selectively programmed by applying different polarities of external bias: positive bias is used for the writing process (HRS→LRS) and negative bias is used for the erasing process (LRS à HRS). Moreover, under time-variation and bending-varying stress, the programmed resistance states were not degraded, which clearly demonstrate that the flexible AD-CBRAM successfully operates as the NVM.

In addition, we reveal the novel film formation mechanism (cushioning effect of the ductile substrate). In the SAED pattern of the bottom region of the composite layer, strong and asymmetric diffraction were detected, implying that large grains (less fractured particles) exist in the bottom region; the STEM image also clearly shows the less fractured particles in the bottom region. As a result, the less fractured particles lead to the porous layer containing voids near the substrate. This effect is due to the difference between the mechanical properties of the substrate (having high ductility) and particle (having high hardness), which is known as the cushioning effect.

These results demonstrated the feasibility of the use of AD method for the fabrication of flexible electronic devices, and it will provide effective guidance for the manufacturing of AD method-based flexible electronic device applications.

## Supplementary information

Applicability of Aerosol Deposition Process for flexible electronic device and determining the Film Formation Mechanism with Cushioning Effects_Supplementary information

## References

[1] Akedo J, Lebedev M (1999). Microstructure and electrical properties of lead zirconate titanate (Pb (Zr52/Ti48) O3) thick films deposited by aerosol deposition method. Jpn. J. Appl. Phys..

[2] Akedo J (2008). Room temperature impact consolidation (RTIC) of fine ceramic powder by aerosol deposition method and applications to microdevices. J. Therm. Spray Technol..

[3] Hanft D (2015). An overview of the aerosol deposition method: Process fundamentals and new trends in materials applications. J. Ceram. Sci. Technol.

[4] Akedo J (2006). Aerosol deposition of ceramic thick films at room temperature: densification mechanism of ceramic layers. J. Am. Ceram. Soc..

[5] Kim HJ (2012). Residual Stress Relief in Al_2_O_3_–Poly-Tetra-Fluoro-Ethylene Hybrid Thick Films for Integrated Substrates Using Aerosol Deposition. J. Nanoelectron. Optoelectron..

[6] Park JH, Akedo J, Nakada M (2006). Surface plasmon resonance in novel nanocomposite gold/lead zirconate titanate films prepared by aerosol deposition method. Jpn. J. Appl. Phys..

[7] Schindler C, Valov I, Waser R (2009). Faradaic currents during electroforming of resistively switching Ag–Ge–Se type electrochemical metallization memory cells. Phys. Chem. Chem. Phys.

[8] Liang JG (2017). Preparation of Ultrasensitive Humidity-Sensing Films by Aerosol Deposition. ACS Appl. Mater. Interfaces.

[9] Lee DW, Kim HJ, Kim YH, Yun YH, Nam SM (2011). Growth Process of α‐Al_2_O_3_ Ceramic Films on Metal Substrates Fabricated at Room Temperature by Aerosol Deposition. J. Am. Ceram. Soc..

[10] Kim HK, Lee SH, Lee SG, Lee YH (2015). Densification mechanism of BaTiO 3 films on Cu substrates fabricated by aerosol deposition. Electron. Mater. Lett.

[11] Kim, D. W. *et al*. Multi Level Operation of CuO Based Cbram with Cute Electrode. In Meeting Abstracts No. 16, 768–768 (2015, July).

[12] Aga FG (2016). Retention modeling for ultra-thin density of Cu-based conductive bridge random access memory (CBRAM). AIP Adv..

[13] Song MJ, Kwon KH, Park JG (2017). Electro-Forming and Electro-Breaking of Nanoscale Ag Filaments for Conductive-Bridging Random-Access Memory Cell using Ag-Doped Polymer-Electrolyte between Pt Electrodes. Sci. rep..

[14] Yang Y (2012). Observation of conducting filament growth in nanoscale resistive memories. Nat. Commun.

[15] Kwon K (2015). Nanoscale CuO solid-electrolyte-based conductive-bridging-random-access-memory cell operating multi-level-cell and 1selector1resistor. J. Mater. Chem. C.

[16] Celano, U. *et al*. Conductive-AFM tomography for 3D filament observation in resistive switching devices. In *Proceedings of the IEEE International Electron Device Meeting* (2013).

[17] Celano U (2014). Three-dimensional observation of the conductive filament in nanoscaled resistive memory devices. Nano lett..

[18] Zhao, Y. D. *et al*. Atomic Monte-Carlo simulation for CBRAM with various filament geometries. In: *International Conference on Simulation of Semiconductor Processes and Devices (SISPAD), IEEE*., pp. 153–156, 2016.

[19] Ryu HS (2013). Corrosion protection performance of YSZ coating on AA7075 aluminum alloy prepared by aerosol deposition. J. Electrochem. Soc..

[20] Kim SW (2018). Fabrication of xenogeneic bone-derived hydroxyapatite thin film by aerosol deposition method. Appl. Surf. Sci..

[21] Lee DW (2017). Brushite ceramic coatings for dental brace brackets fabricated via aerosol deposition. Ceram. Int..

[22] Hsiao CC (2014). A rapid process for fabricating gas sensors. Sensors..

[23] Sahner K (2009). Assessment of the novel aerosol deposition method for room temperature preparation of metal oxide gas sensor films. Sensors Actuators B..

[24] Ryu J (2011). In-plane impedance spectroscopy in aerosol deposited NiMn_2_O_4_ negative temperature coefficient thermistor films. J. Appl. Phys.

[25] Choi JJ (2012). Low-temperature preparation of dense (Gd, Ce) O_2−δ_–Gd_2_O_3_ composite buffer layer by aerosol deposition for YSZ electrolyte-based SOFC. Int. J. Hydrogen Energy.

[26] Bae H (2015). Low-temperature fabrication of protonic ceramic fuel cells with BaZr_0.8_Y_0.2_O_3−δ_ electrolytes coated by aerosol deposition method. Int. J. Hydrogen Energy.

[27] Nakada M (2009). Lanthanum-modified lead zirconate titanate electro-optic modulators fabricated using aerosol deposition for LSI interconnects. Jpn. J. Appl. Phys..

[28] Iwanami M (2007). Ultra small electro-optic field probe fabricated by aerosol deposition. IEICE Electro. express.

[29] Oh JM, Nam SM (2009). Possibility of BaTiO_3_ thin films prepared on cu substrates for embedded decoupling capacitors by an aerosol deposition method. J. Ceram. Process..

