# Supplementary material for: Applicability of Aerosol Deposition Process for flexible electronic device and determining the Film Formation Mechanism with Cushioning Effects
Source: Sci Rep. 2019 Feb 15;9:2166. doi: 10.1038/s41598-019-38477-y (PMC6377626; doi:10.1038/s41598-019-38477-y)
Supplement: Supplementary file 1 — Applicability of Aerosol Deposition Process for flexible electronic device and determining the Film Formation Mechanism with Cushioning Effects_Supplementary information [file 41598_2019_38477_MOESM1_ESM.pdf]

Applicability of Aerosol Deposition Process for flexible electronic device and determining the Film Formation Mechanism with Cushioning Effects

Chuljun Lee, Myung-Yeon Cho, Myungjun Kim, Jiyun Jang, Yoonsub Oh, Kihoon Oh, Seunghyun Kim, Byungwook Park, Byungkwan Kim, Sang-Mo Koo, Jong-Min Oh* & Daeseok Lee*

Supplementary information

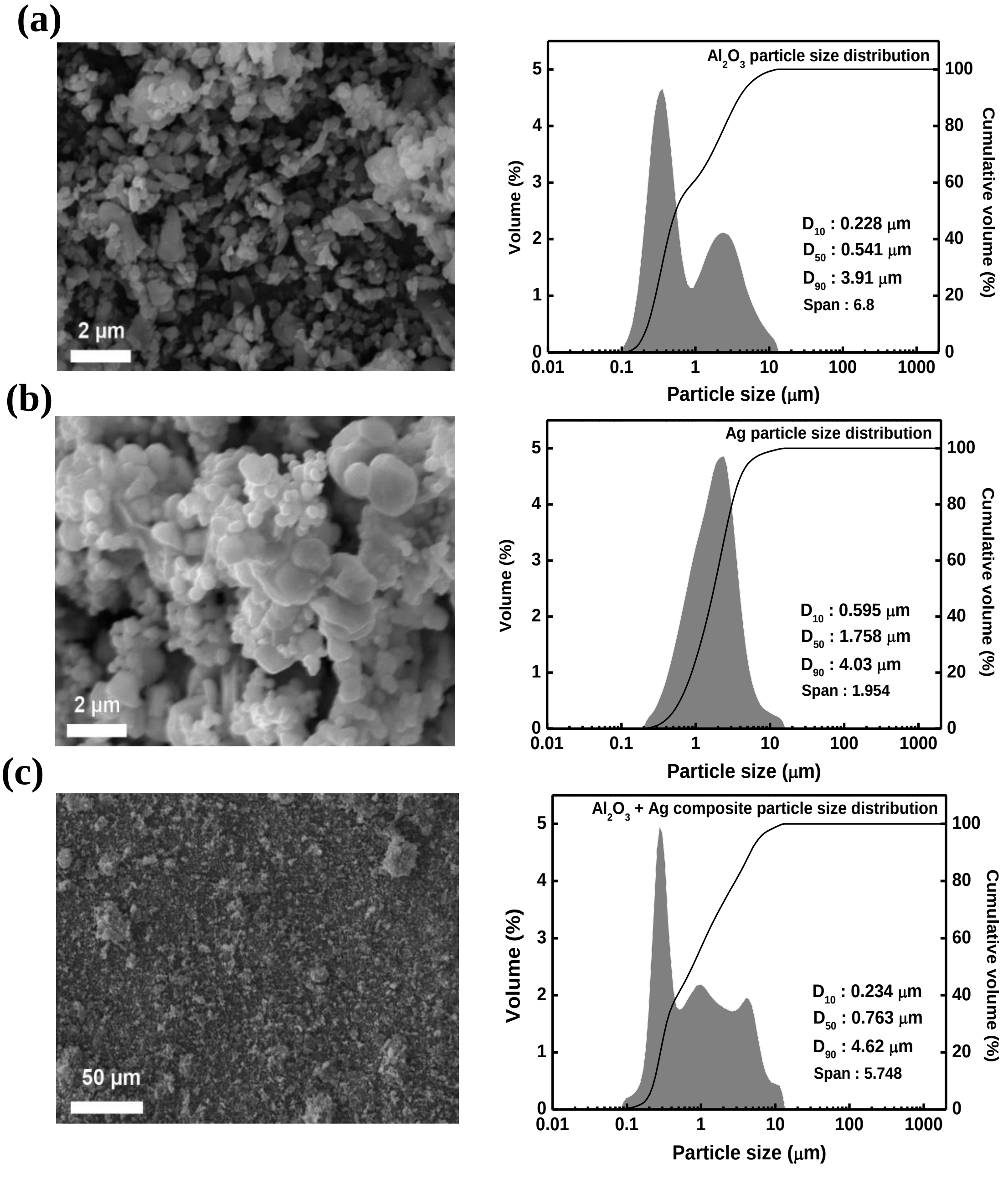

(d)

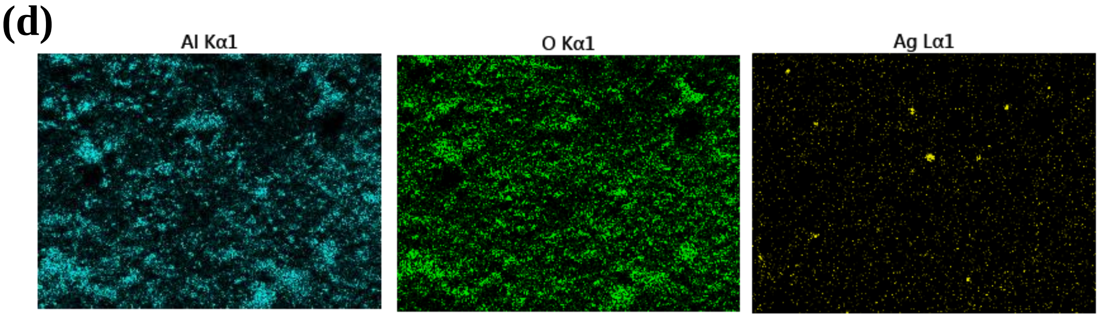

Supplementary Figure S1: SEM image and particle size distribution for utilized (a) Al_2O_3 powder (b) Ag powder and (c) Al_2O_3/Ag composite powder. (d) EDS mapping analysis in (c); it shows that Ag particles are distributed in the composite powder.

Deposition conditions	
**Starting composite powder**	Al_2O_3 (99.8 wt%) + Ag (0.2 wt%)
**Substrate**	Aluminum (flexible)
**Base pressure**	0.07 [Torr]
**Carrier gas**	He
**Consumption of carrier gas**	14-16L/min
**Scan number**	5
**Size of nozzle orifice**	10 x 0.4 mm^2
**Deposition temperature**	Room temperature
**Annealing process**	x

Supplementary Table S1: Detailed deposition conditions for Al_2O_3/Ag composite layer.

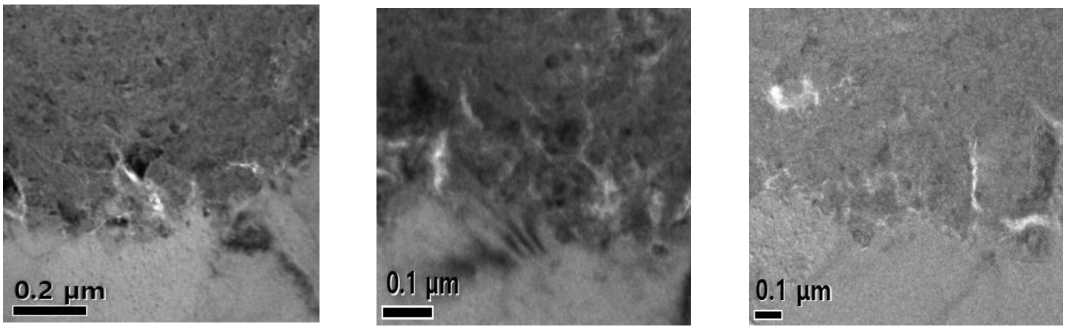

Supplementary Figure S2: Cross-sectional TEM images of composite layer near the aluminum substrate show that voids are distributed in the bottom region of the composite layer. Additionally, the dented layers formed by the collision of the accelerated particles are distributed at the interface of the substrate and composite layer.

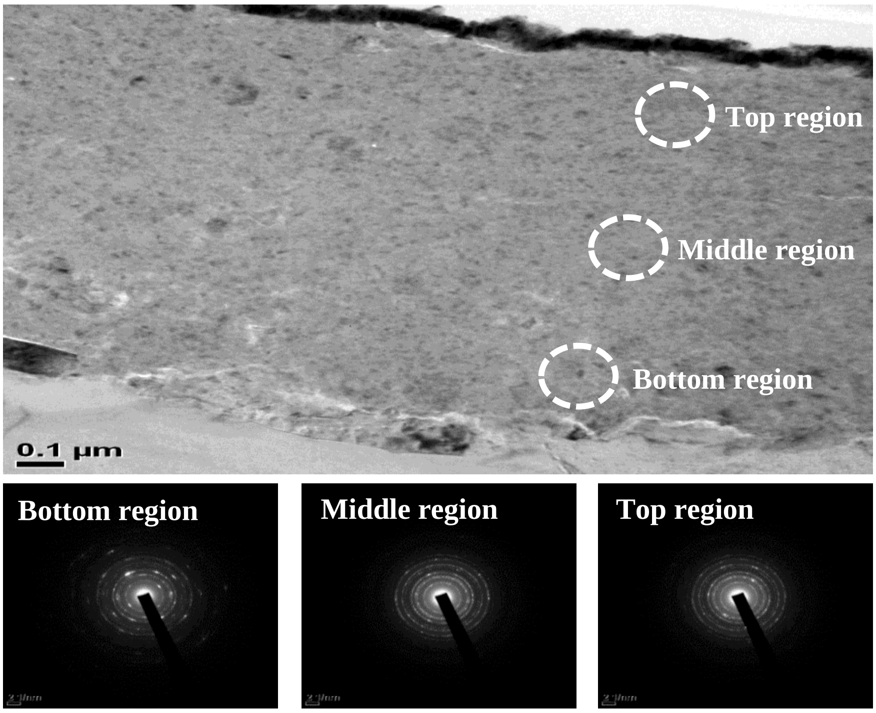

Supplementary Figure S3: For the first-deposited composite layer (bottom region), stronger diffraction is detected than post-deposited layer (middle ~ top region). This result shows that the difference in diffraction strength can be caused by difference of particles size; the relatively large grains are located in the bottom region.

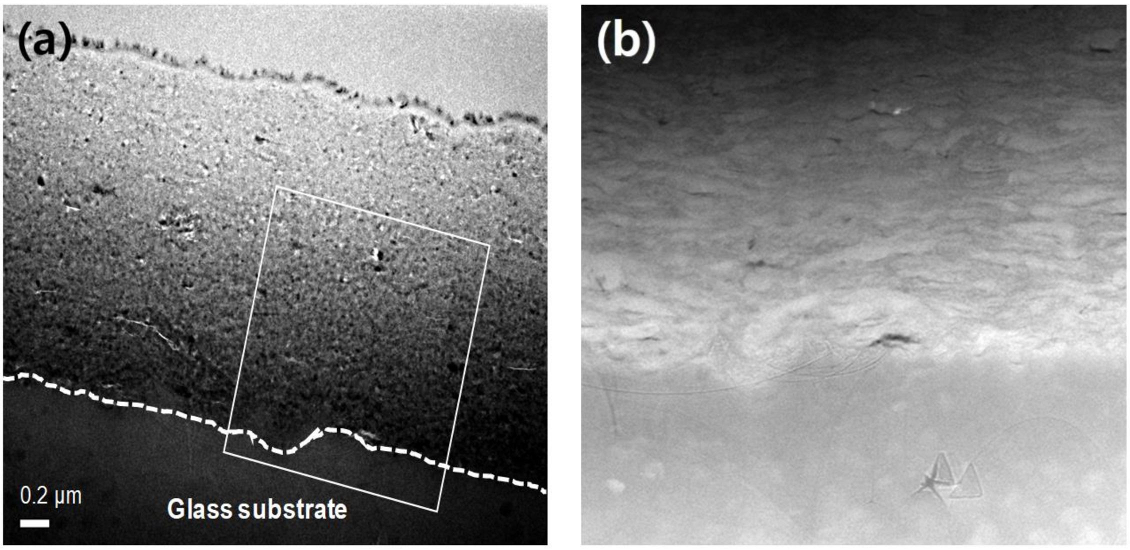

Supplementary Figure S4: Cross-sectional (a) TEM image and (b) STEM image for composite layer which is deposited on the glass substrate having hardness. It shows the bottom region has more dense than upper region, as well as un-fractured particles not distributed at the bottom region.
